# Late decompressive craniectomyafter traumatic brain injury: neurological outcome at 6 months after ICU discharge

**DOI:** 10.1186/1752-2897-6-8

**Published:** 2012-08-06

**Authors:** Giovanni Cianchi, Manuela Bonizzoli, Giovanni Zagli, Simona di Valvasone, Simona Biondi, Marco Ciapetti, Lucia Perretta, Furio Mariotti, Adriano Peris

**Affiliations:** 1 Anesthesia and Intensive Care Unit of Emergency Department, Careggi Teaching Hospital, Largo Brambilla 3, 50139 Florence, Italy; 2 Postgraduate School of Anesthesia and Intensive Care, Careggi Teaching Hospital, Largo Brambilla 3, 50139, Florence, Italy; 3 Department of Neurosurgery, Careggi Teaching Hospital, Largo Brambilla 3, 50139, Florence, Italy

## Abstract

**Introduction:**

The choice of optimal treatment in traumatic brain injured (TBI) patients is a challenge. The aim of this study was to verify the neurological outcome of severe TBI patients treated with decompressive craniectomy (early < 24 h, late > 24 h), compared to conservative treatment, in hospital and after 6-months.

**Methods:**

A total of 186 TBI patients admitted to the ICU of the Emergency Department of a tertiary referral center (Careggi Teaching Hospital, Florence, Italy) from 2005 through 2009 were retrospectively studied. Patients treated with decompressive craniectomy were divided into 2 groups: “early craniectomy group” (patients who underwent to craniectomy within the first 24 hours); and “late craniectomy group” (patients who underwent to craniectomy later than the first 24 hours). As a control group, patients whose intracranial hypertension was successfully controlled by medical treatment were included in the “no craniectomy group”.

**Results:**

Groups included 41 patients who required early decompressive craniectomy, 21 patients treated with late craniectomy (7.7 days after trauma, on average), and 124 patients for whom intracranial hypertension was successfully controlled through conservative treatment. Groups were comparable in age and trauma/critical illness scores, except for a significantly higher Marshall score in early craniectomized patients. The Glasgow Outcome Scale was comparable between groups at ICU, at the time of hospital discharge and at 6 months.

**Conclusions:**

In our sample, a late craniectomy in patients with refractory intracranial hypertension produced a comparable 6-months neurological outcome if compared to patients responder to standard treatment. This data must be reproduced and confirmed before considering as goal-treatment in refractory intracranial hypertension.

## Introduction

The need for intracranial pressure control is the primary goal in severe traumatic brain injured (TBI) patients. Besides the direct injury, secondary insult due to post-traumatic increase of intracranial pressure or decrease in cerebral perfusion pressure are well recognized as causes of increasing mortality and morbidity [1-6].

In case of clinical and/or CT-scan signs of relevant acute space-occupying lesions, early decompressive craniectomy can efficiently control the increase of intracranial pressure and the development of secondary damage. However, surgical management is also affected by complications such as secondary hematoma or regional ischemia due to herniation through the operculum [7]. These features render it difficult to decide whether or not to perform a decompressive craniectomy at alater phase in case of intracranial hypertension unresponsiveness to medical treatment. In this regard, the effective role of decompressive craniectomy compared with barbiturate coma in raised refractory intracranial hypertension is still under evaluation by the ongoing RESCUEicp study [8].

In the present study we show our finding in evaluating both intra-ICU and 6-months outcome of severe TBI patients treated with early craniectomy (<24 hours) and late craniectomy (>24 hours).

## Methods

### Patient selection and data management

This is a retrospective cohort study which includes patients with severe head trauma (Glasgow Coma Scale <9) admitted to the 10-bed ICU of the Emergency Department of a tertiary referral center (Careggi Teaching Hospital, Florence, Italy) from 2005 through 2009. For each patient, data from institutional ICU and follow-up databases (FileMaker Pro, FileMaker, Inc, USA), and from the Italian Group for the Evaluation of Interventions in Intensive Care Medicine database (GiViTI Margherita Project, Istituto Mario Negri, Bergamo, Italy), were collected: age; gender; Marshall score [9]; Glasgow Coma Scale (GCS) at admission, at ICU discharge and at hospital discharge; Injury Severity Score (ISS); RTS: Revised Trauma Score; Trauma Injury Severity Score (TRISS); Simplified Acute Physiology Score (SAPS) II; ICU and Hospital Length of Stay (LOS); Glasgow Outcome Scale (GOS) at 6 months; ICU and hospital mortality. The Institutional Internal Review Board approved the study, and informed consent for data publication were obtained from the patients or their relatives.

As an objective index of ability to function after injury, the Glasgow Outcome Scale (GOS) was used [10]. The 6-months assessment is part of the post-intensive institutional follow-up protocol, and is performed by independent staff. The score was assigned as follows:

1: dead;

2: vegetative state; awake but not aware; does not interact in any cognitive way with the environment; does not fixate or follow with eyes; vegetative functions preserved;

3: severe disability; able to follow commands but cannot live independently; requires support for activities of daily living;

4: moderate disability; able to participate in activities of daily living, but work and social life are compromised because of mental or physical disability;

5: good recovery; able to return to work or school.

For analysis, patients treated with decompressive craniectomy were divided in 2 groups:

1) “Early craniectomy group” (patients who underwent to craniectomy within the first 24 hours after hospital admission based on Marshall score);

2) “Late craniectomy group” (patients who underwent to craniectomy later than the first 24 hours after hospital admission due an intracranial pressure above 30 mmHg refractory to medical treatment).

As control group, patients whose intracranial hypertension was successfully controlled by medical treatment were included in the “no craniectomy group”.

### ICU patient care and intracranial pressure management

Patients with traumatic brain injury were managed by our ICU protocol by a group of certified trauma/critical care intensivists using current, evidence-based practice management guidelines [5]. Treatment goal was the maintenance of cerebral perfusion pressure above 60 mmHg and intracranial pressure below 20 mmHg. Traumatic brain injury evolution was followed up by CT-scan at 6 and 24 hours after event, and later if clinically indicated.

All patients who did not underwent to immediate neurosurgical decompressive procedure after hospital admission were monitored by intra-ICU intracranial probes (Camino®, Integra Neurosciences, Plainsboro, NJ) positioning performed by the intensivist on duty. In case of pre-ICU neurosurgical procedure, intracranial probe (Codman® ICP probe, Raynham, MA) were positioned by neurosurgeon. Following internal hospital protocol, ventricular drainage is not provided for treatment of intracranial hypertension, except in selected cases. In the present study, none of patients had cerebrospinal fluid drainage.

Patients were positioned with 25–30 degrees inclination head-up. Adequate pain control and sedation were provided by fentanyl, propofol and/or midazolam. In case of refractory intracranial pressure above 30 mmHg, barbiturate coma (thiopentone sodium) was achieved under continuous EEG monitoring, and dose titration was made as soon as possible based on burst suppression maintenance.

Normovolemia was assessed by isotonic infusions based on central venous pressure and central venous oxygen saturation monitoring. Hemodynamic support (norepinephrine/dobutamine) was achieved with vasoactive/inotropic agents (norepinephrine/dobutamine) in order to maintain cerebral perfusion pressure above 60 mmHg. Serum osmolarity was maintained at 320 mOsm/l and serum sodium within 145–155 mEq/L.

Patient temperature was monitored and controlled by the intracranial probe transducer. In case of hyperthermia, brain temperature was controlled with antipyretic drugs or, in case of unresponsiveness, by intravascular cooling system (Alsius corp., Irvine, California, USA). Early nutritional support, peptic ulcer prophylaxis and glycaemic control were guaranteed. Deep venous thrombosis prophylaxis was achieved for the first 72 hours by using sequential compression devices after ultrasound control of vein districts by the intensivist on duty [11]. Subsequently, low-molecular weight heparin prophylaxis was provided.

During ICU stay, all patients included in this study were tracheostomized by percutaneous dilatational bedside procedure [12]. Patients were ventilated to obtain a PaCO_2_ level between 35–40 mmHg using protective ventilation modes. For intracranial pressure measurements greater than 20 mmHg, controlled hypocarbia (>25 mmHg) was achieved by jugular venous oximetry monitoring (maintained above 60%). If needed, mannitol or hypertonic saline (3%) was instituted.

### Statistical analysis

GraphPad Prism 5 (GraphPad Software Inc., San Diego, CA) was used for statistical analysis. Continuous variables were analyzed with Kruskal-Wallis test and Dunn's multiple comparison test post hoc. Categorical data were examined using Chi-square test and Fisher's exact text (95% confidence interval). Results were expressed as mean ± standard deviation (SD). P values were considered significant if less than 0.05.

## Results

In all, 186 patients were studied (Table 1). Patients who needed decompressive craniectomy (early or late, N = 62) did not differ significantly in demographic, clinical or outcome parameters from patients whose intracranial pressure was controlled by medical treatments (N = 124), except for a significantly higher six-month mortality rate (46.8% vs 29%; P = 0.02). No intracranial probe infection-related or secondary hemorrhages occurred in overall population.

**Table 1:** Baseline characteristics and outcome parameters of patients underwent to craniectomy (early and late) and patients treated with medical therapy

	**Overall**	**Craniectomy**	**Nocraniectomy**	**P value**
**Number**	186	62	124	
**Male sex, % (N)**	76.9% (146)	75.8% (47)	65.3% (81)	0.85
**Age (years)**	45 ± 20.4	41.1 ± 18.7	47 ± 21	0.07
**ISS**	32.1 ± 11.5	33.1 ± 10.2	31.7 ± 12.1	0.17
**RTS**	5.5 ± 1.5	5.5 ± 1.4	5.5 ± 1.6	0.99
**TRISS**	63.1 ± 27	63.1 ± 25.8	63.1 ± 27.7	0.76
**GCS at admission**	7.4 ± 3.5	7.5 ± 3.3	7.4 ± 3.6	0.60
**SAPS II**	45.4 ± 13.3	44.9 ± 11.6	46.2 ± 14.4	0.88
**ICU LOS**	19.1 ± 10	20.5 ± 10.2	18.4 ± 10	0.22
**ICU mortality, % (N)**	25.8% (48)	29% (18)	24.2% (30)	0.48
**GCS at ICU discharge**	7.8 ± 4	7.2 ± 3.5	8 ± 4.3	0.22
**Total hospitalmortality, % (N)**	31.2% (58)	35.5% (22)	29% (36)	0.40
**Hospital LOS**	42.6 ± 21.9	47.1 ± 23.6	40.2 ± 20.6	0.07
**GCS at hospital discharge**	10.9 ± 4	10.6 ± 4	10.9 ±4	0.45
**GOS at 6 months**	3.3 ± 1.2	3.2 ± 1.3	3.6 ± 0.9	0.29
**Total 6-monthsmortality, % (N)**	34.9% (65)	46.8% (29)	29% (36)*	0.02

Continuous variables are expressed as mean ± standard deviation. Percentages are referred to the total population of each group. Statistical analysis: Mann–Whitney test (Craniectomy vs No craniectomy)andChi square test. P value was considered significant if <0.05.

*AIS*: Abbreviated Injury Scale; *GCS*: Glasgow Coma Scale; *GOS*: Glasgow Outcome Scale; *LOS*: Length of Stay; *ISS*: Injury Severity Score; *RTS*: Revised Trauma Score; *SAPS*: Simplified Acute Physiology Score; *TRISS*: Trauma - Injury Severity Score.

*P < 0.05.

In order to understand the difference between patients who underwentearly and late decompressive craniectomy, a subgroup analysis was conducted (Table 2). Subgroups were not numerically homogenous, including 41 patients who required early decompressive craniectomy, 21 patients treated with late craniectomy, and 124 patients in whom intracranial hypertension was successfully controlled with conservative intensive care.

**Table 2:** Comparison between subgroups of patients based on treatment

	**Early craniectomy**	**Late craniectomy**	**No craniectomy**	**P value**
**Number**	41	21	124	
**Male sex, % (N)**	70.7% (29)	76.2% (16)	65.3% (81)	0.99
**Age (years)**	43.8 ± 17.9	35.7 ± 19.4	47 ± 21	0.07
**ISS**	33.3 ± 10.9	32.7 ± 8.9	31.7 ± 12.1	0.38
**RTS**	5.7 ± 1.5	5.3 ± 1.3	5.5 ± 1.6	0.52
**TRISS**	63.4 ± 28.4	62.6 ± 20.4	63.1 ± 27.7	0.84
**GCS at admission**	7.7 ± 3.5	7.2 ± 3	7.4 ± 3.6	0.85
**Marshall score**	3.2 ± 0.8*	2.4 ± 0.8	2.7 ± 0.9	0.02
**SAPS II**	46.1 ± 12.5	42.4 ± 9.2	46.2 ± 14.4	0.58
**ICU LOS**	16.6 ± 9.1	28 ± 7.6***	19.1 ± 10	0.001
**ICU mortality, % (N)**	29.3% (12)	28.6% (6)	24.2% (30)	0.91
**GCS at ICU discharge**	7.1 ± 3.5	7.5 ± 3.5	8 ± 4.3	0.42
**Total hospitalmortality, % (N)**	36.6% (15)	33.3% (7)	29% (36)	0.65
**Hospital LOS**	43.1 ± 24.7	54.2 ± 21.2*	40.2 ± 20.6	0.02
**GCS at hospital discharge**	10.7 ±4.3	10.5 ±3.5	11 ± 4	0.74
**GOS at 6 months**	3.3 ± 1.4	3.00 ± 1.1	3.6 ± 0.9	0.71
**Total 6-monthsmortality, % (N)**	48.8% (20)	42.9% (9)	29% (36)*	0.02

Continuous variables are expressed as mean ± standard deviation. Percentages are referred to the total population of each group. Statistical analysis: Kruskal-Wallis test with Dunn's multiple comparison test andChi square test. P value was considered significant if <0.05.

*AIS*: Abbreviated Injury Scale; *GCS*: Glasgow Coma Scale; *GOS*: Glasgow Outcome Scale; *LOS*: Length of Stay; *ISS*: Injury Severity Score; *RTS*: Revised Trauma Score; *SAPS*: Simplified Acute Physiology Score; *TRISS*: Trauma - Injury Severity Score.

*P < 0.05.

***P < 0.001.

As shown in Table 2, groups were comparable in age and trauma/critical illness scores, except for a significantly higher Marshall score in early craniectomized patients (3.2 vs 2.4; P = 0.02). In the late craniectomy group, surgical decompression was performed after 7.7 ± 5.1 days from injury, on average.

Overall, in patients who underwent craniectomy, perioperative complications were: ipsilateral hemorrhagic extension of lesion (33%), herniation through operculum (17%), ipsilateral non-hemorrhagic extension of lesion (10%), and contralateral hematoma (8%). Ipsilateral hemorrhagic extension of lesion was significantly more frequent in early decompressed patients (46% vs 14%, p < 0.05). Late craniectomized patients showed a significantly higher ICU and total hospital LOS than the other groups. Intra-ICU and hospital mortality rates were similar in all groups. Also, neurological scores were comparable between groups both at ICU and hospital discharge (Table 2).

Groups did not differ significantly in GOS at 6 months. The most frequent disabilities, distributed equally among groups, were: memory dysfunctions (15%), difficulty in speech-language (12%), epilepsy (12%), and headache (12%). Despite similar neurological outcome, patients who did not require surgical intervention showed a significantly lower mortality rate at 6 months after trauma (Table 2).

## Discussion

The main finding in our experience is the comparable neurological outcome in patients responder to medical therapy and patients with intracranial pressure who underwent to late decompressive craniectomy. Our results are mainly limited by the retrospective nature of the study, and the lack of a control group of patients who are non-responders to medical therapy not treated with late craniectomy is a consequence of the study design, even if we wonder about the ethics of including such a group in a randomized controlled trial. The sample size of the three groups is different, especially regarding the late craniectomized patients: this feature must be taken into consideration in reading the results. Finally, concerning the craniectomy-related complications, our data did not show any difference between early and late treated patients, but the small study population does not allow investigation into this important aspect.

Recently, Cooper and co-workers published their results of a multicenter trial in which a group of patients (N = 73) underwent to an early (<72 hours) bifrontotemporoparietal decompressive craniectomy was compared to a standard care (N = 82) group (DECRA study) [13]. Despite the better control in intracranial pressure, shorter duration of mechanical ventilation and shorter ICU length of stay observed in the intervention group, patients treated with decompressive craniectomy had a more unfavorable outcome at 6 months. The study of Cooper and co-workers was randomized and multicenter, even if the very long duration (about 8 years) and the relatively small sample size of groups limits the strength of results, but, more important in regard of the present study, the DECRA study was designed to evaluate the effects of early decompressive craniectomy, so our data in regard of a rescue late craniectomy cannot be compared with the results of Cooper and co-authors.

A Cochrane review failed to find evidence in support of routine use of late decompressive craniectomy in patients with TBI, except in pediatric patients [14]. However, the Authors stated that the results of non-randomized trials suggest that a late craniectomy may have a therapeutic role when full intensive medical treatment has failed to control intracranial hypertension.

Eberle and co-authors performed a retrospective analysis on 43 patients who underwent decompressive craniectomy in the absence of a space occupying haemorrhage within the first 24 hours after admission, showing that the early surgical treatment was effective in decreasing intracranial pressure and increasing cerebral perfusion pressure in survivors compared to non-survivors [15]. These promising results were limited by the study design and the absence of a control group. A second study by Weiner and co-workers showed that decompression craniectomy performed at an average of 2.8 days after admission was effective in decreasing the predicted mortality based on intracranial pressure level and the intensity of care needed [16]. This paper was not designed with a control group, so no information concerning optimal timing of decompressive craniectomy can be found, and no neurological outcome was included, but the data showed by Weiner and colleagues suggesting the favorable role of an early intervention in patient outcome.

In the light of the available literature, the choice of optimal treatment in severe brain injured patients remains a challenge for clinicians. In our sample, early craniectomy, chosen due to CT-scan image evidence, produced a similar neurological outcome to patients with no emergency surgical indications who resulted as responders to medical treatment (necessarily cerebral perfusion pressure guided), even if the 6-month mortality rate remained higher in patients with a worse Marshall score (Table 2). In our opinion, the main finding of our study is the demonstration that patients who are non-responders to medical treatment, and who have a late decompressive craniectomy, show similar neurological recovery compared with patients withintracranial pressure that could be controlled medically. This may help intensivists and neurosurgeons in their decision making process.

It is not surprising that patients in the late craniectomy groups showed longer ICU and hospital stays, since the surgical intervention was preceded by full medical treatment (almost 8 days on average). In addition, late craniectomy groups presented a mean age slightly lower than the other groups (Table 2). This feature might be retrospectively understood considering the spontaneous hope of physicians that young age may be associated with better responsiveness to medical treatment. At 6 months, patients who did not undergo surgical treatment showed a significantly lower mortality rate than patients of the early and late decompressive groups (29% vs 48.8% and 42.9%, respectively; Table 2), most likely due to the lower time of brain suffering.

## Conclusion

The present study, due to its design and limitations, cannot indicate that late decompressive craniectomy for post-traumatic refractory intracranial hypertension can become the goal-treatment. Moreover, the higher mortality risk observed in surgical patients must be always taken into consideration during the decision-making process. However, the finding that, in our sample, patients who were non-responders to medical treatment showed similar neurological recovery compared with patients with controlled intracranial pressure, may help intensivists and neurosurgeons during the evaluation of the opportunity to proceed to a late decompressive craniectomy in case of failure of medical PIC-guided treatment.

## Abbreviations

AIS: Abbreviated Injury Scale; GCS: Glasgow Coma Scale; GOS: Glasgow Outcome Scale; LOS: Length of Stay; ICU: Intensive Care Unit; ISS: Injury Severity Score; RTS: Revised Trauma Score; SAPS: Simplified Acute Physiology Score; TBI: Traumatic brain injury; TRISS: Trauma - Injury Severity Score.

## Competing interests

The authors declare that they do not have any conflicts of interest.

## Author’s contributions

GC, MB, FM, AP designed the study; GC, MB, GZ, MC, AP reviewed the literature; SdV, LP, FM collected data; GZ and SdV performed statistical analysis; GC, GZ, SdV, AP wrote the draft. All Authors revised the manuscript and approved the final version.

## Author details

^1^Anesthesia and Intensive Care Unit of Emergency Department, Careggi Teaching Hospital, Largo Brambilla 3, 50139 Florence, Italy. ^2^Postgraduate School of Anesthesia and Intensive Care, Careggi Teaching Hospital, Largo Brambilla 3, 50139 Florence, Italy. ^3^Department of Neurosurgery, Careggi Teaching Hospital, Largo Brambilla 3, 50139 Florence, Italy.
